# Intergenerational effects of racism on amygdala and hippocampus resting state functional connectivity

**DOI:** 10.1038/s41598-024-66830-3

**Published:** 2024-07-24

**Authors:** T. R. A. Kral, C. Y. Williams, A. C. Wylie, K. McLaughlin, R. L. Stephens, W. R. Mills-Koonce, R. M. Birn, C. B. Propper, S. J. Short

**Affiliations:** 1 Center for Healthy Minds, University of Wisconsin –Madison, Madison, WI USA; 2 Department of Psychiatry, University of Wisconsin –Madison, Madison, USA; 3 Department of Counseling Psychology, University of Wisconsin –Madison, Madison, USA; 4 Frank Porter Graham Child Development Institute, University of North Carolina at Chapel Hill, Chapel Hill, USA; 5 Department of Psychology and Neuroscience, University of North Carolina at Chapel Hill, Chapel Hill, USA; 6 Department of Psychiatry, University of North Carolina at Chapel Hill, Chapel Hill, USA; 7 School of Education, University of North Carolina at Chapel Hill, Chapel Hill, USA; 8 School of Nursing, University of North Carolina at Chapel Hill, Chapel Hill, USA; 9 Department of Educational Psychology, University of Wisconsin –Madison, Madison, USA

**Keywords:** Racism, Trauma, Amygdala, Hippocampus, Functional connectivity, Psychology, Neuroscience, Social neuroscience

## Abstract

Racism is an insidious problem with far-reaching effects on the lives of Black, Indigenous, and People of Color (BIPOC). The pervasive negative impact of racism on mental health is well documented. However, less is known about the potential downstream impacts of maternal experiences of racism on offspring neurodevelopment. This study sought to examine evidence for a biological pathway of intergenerational transmission of racism-related trauma. This study examined the effects of self-reported maternal experiences of racism on resting state functional connectivity (rsFC) in n = 25 neonates (13 female, 12 male) birthed by BIPOC mothers. Amygdala and hippocampus are brain regions involved in fear, memory, and anxiety, and are central nodes in brain networks associated with trauma-related change. We used average scores on the Experiences of Racism Scale as a continuous, voxel-wise regressor in seed-based, whole-brain connectivity analysis of anatomically defined amygdala and hippocampus seed regions of interest. All analyses controlled for infant sex and gestational age at the 2-week scanning session. More maternal racism-related experiences were associated with (1) stronger right amygdala rsFC with visual cortex and thalamus; and (2) stronger hippocampus rsFC with visual cortex and a temporo-parietal network, in neonates. The results of this research have implications for understanding how maternal experiences of racism may alter neurodevelopment, and for related social policy.

## Introduction

Racism is a public health crisis and a fundamental cause of health disparities and inequities among Black, Indigenous, and People of Color (BIPOC)^1,2^. By racism, we refer to a social system whereby individuals are divided into ‘races’ and treated differently (i.e., with unequal power), such that the group in power (e.g., “White”) systematically discriminates against other racial groups through institutional policies and practices. This system of race-based discrimination can be embodied through attitudes, behaviors, laws, and norms^3^, and racist cultural practices can be both shaped by and serve to support racism. In addition to having detrimental effects on physical health^4^, experiences of racism can have severe negative effects on mental health^5,6^. Despite these known associations, research examining the downstream effects of maternal, racism-related trauma on neonatal neurodevelopment remains scarce. The current study sought to address this question by examining neural mechanisms that may underly the intergenerational transmission of the negative impacts of racism, biologically. Intergenerational transmission in this context refers to biological changes in parents that alter infant biology and/ or outcomes via changes during the prenatal period^7,8^.

A well-established body of research among adults indicates that experiences of racism can be associated with severe negative impacts on individuals’ mental health, including anxiety, depression, stress^6^, symptoms of post-traumatic stress disorder (PTSD)^9^, and other psychopathologies^10,11^. Research in psychiatry and public health supports the theory that the cumulative load of trauma can contribute to the intergenerational transmission of stress^12,13^, and these stressors are intensified for individuals with intersectional identities, such as black women who are exposed to race- and gender-based discrimination.

The potential for intergenerational transmission of biological impacts of discrimination to neonates was demonstrated in a separate study. Experiences of discrimination among a sample of primarily Hispanic, teenage mothers were associated with stronger amygdala resting state functional connectivity (rsFC) to the fusiform visual cortical area, and weaker connectivity to medial prefrontal cortex (MPFC)^14^. The brain networks implicated in this prior research overlap with regions with known roles in vigilance and threat processing, e.g. stronger amygdala–visual cortex rsFC^14^. In a sample of 31 healthy adults, stronger amygdala-visual cortex connectivity was associated with general increased vigilance and anxiety during the presentation of novel visual stimuli^15^, and a study in adults with PTSD showed associations between ventral visual stream connectivity, amygdala connectivity, and symptoms^16^. Moreover, mechanistic research indicates heightened sensitivity and decreased selectivity of amygdala response during acute stress, combined with heightened response in visual cortex, is consistent with the state of high vigilance^17^.

Recent research also shows that experiences of racism in adults are associated with stronger activation and connectivity of brain regions involved in vigilance, attention, and emotion. One study found that more experiences of racial discrimination among Black adults was associated with increased resting state connectivity in nodes of the salience network involved in vigilance, including the amygdala^18^. Another study reported that Black women who experienced more instances of racism had higher brain activation in ventromedial prefrontal cortex and occipital cortex—brain regions associated with emotion regulation and visual attention, respectively, while viewing trauma-related images, and heightened neural response to threat-related stimuli^19^. Additionally, discrimination more generally has been associated with increased amygdala activity and connectivity^20^. Amygdala and hippocampus are involved in vigilance, threat detection, and fear learning in adults^21^ and infants^22^, and have altered function and connectivity in individuals with PTSD^23–25^. Taken together, these findings suggest that higher levels of racism-related trauma may result in alterations in the brain similar to those found in individuals suffering from PTSD, in adults.

There is a growing body of research examining neonatal brain connectivity, which provides evidence that the functional dynamics of amygdala and hippocampus show patterns that mirror their eventual functional dynamics in adults, already during this initial stage of life. Amygdala rsFC was shown to have positive correlations with limbic and subcortical structures, and negative correlations with cortical structures, in healthy neonates^26^, similar to the rsFC patterns of the amygdala in adults^27^. Moreover, this study also showed that neonatal amygdala rsFC was associated with internalizing symptoms at 2 years of age. The patterns of specific symptoms and the regions to which amygdala connectivity was altered in young children, were consistent with the pathophysiology observed in adults related to depression, anxiety, and/ or behavioral inhibition^26^. Similarly, alterations in offspring hippocampal structure and function are consistently reported in animal and human studies of prenatal distress (e.g., stress, anxiety, depression)^28–31^. The neural circuitry of anxiety and behavioral inhibition largely overlaps the neural pathways governing the neuroendocrine stress response which may explain alterations in rsFC associated with both the amygdala and hippocampus^32^.

This separate line of research indicates evidence for pathways by which maternal mental health during the prenatal period may impact the neonatal brain and infant behavior. Human and animal studies point to a direct relationship between maternal prenatal stress and adverse consequences for infant neurodevelopment and mental health, possibly through increased exposure to glucocorticoids in utero^33^. The hippocampus contains high levels of glucocorticoid receptors that regulate the release and homeostasis of glucocorticoid hormones in response to psychological and physiological stressors^34^. Consequently, the hippocampus is particularly sensitive to prolonged or prenatal stressors^35^. Worse maternal prenatal mental health has been associated with impaired structural neurodevelopment, including smaller hippocampal volumes and reduced cortical folding, and altered neurochemistry^36^. Behavioral research found that maternal prenatal depression was associated with heightened fearfulness during infancy^37^, indicating potential downstream effects of heightened glucocorticoid exposure in utero, given the role of elevated glucocorticoids in fear generalization in mouse models^38^.

Research showing associations between maternal prenatal mental health and infant outcomes also provides evidence for pathways by which maternal prenatal stressors, including the negative impacts of racism, may be perpetuated intergenerationally. In fact, a few recent studies have shown associations between prenatal stress and alterations in amygdala and hippocampal functional connectivity^39–43^. However, it is unclear if neonates demonstrate similar neurodevelopmental adaptations when pregnant people have experienced racism, as with other forms of stress, psychopathology, and discrimination, as described above.

In the current study, we tested a potential intergenerational biological pathway by examining whether maternal experiences of racism, reported prenatally, were associated with differences in neonatal functional brain network connectivity during the resting state. We focused our region-of-interest (ROI) analysis on the amygdala and hippocampus, given their roles in trauma, fear, and anxiety, and based on results from prior work showing alterations in neonatal amygdala connectivity in relation to maternal experiences of discrimination^14^. We hypothesized that maternal experiences of racism would be associated with increased amygdala and hippocampus resting state functional connectivity (rsFC) with regions involved in affective processing and emotion regulation, such as medial prefrontal cortex (mPFC).

## Methods and materials

All procedures were approved by UNC–Chapel Hill’s Institutional Review Board (IRB #17-1914); all adult participants provided written, informed consent for themselves and their child prior to data collection and were given monetary compensation for their participation. All research was performed in accordance with the Declaration of Helsinki.

### Participants

Data came from the Brain and Early Experience Study, a prospective longitudinal study investigating the effects of early experience on child development (Mills-Koonce et al., 2022). Pregnant women who resided within a 50-mile radius of UNC–Chapel Hill were identified from medical records, and through flyers, emails, and online advertisements. Potential participants were excluded if they were younger than 18 years of age, did not speak primarily English at home and/or were not experiencing a singleton pregnancy. The sample in this study represents a subsample from a larger study that was examining home and environmental factors that influence the emergence of executive function. Language interactions were one of the focal variables of the larger study and thus, as a first step, we could not include bi- or tri-lingual language learners due to the measurement programs and statistical power needed for the primary study questions.

Following birth, infants were considered as participants and mother-infant dyads were officially enrolled into the study if the infant was born at 36 weeks and 4 days gestational age or older, 5.5 pounds or heavier, not medically fragile, and did not have any medical devices implanted that would preclude infants’ ability to participate in the magnetic resonance imaging (MRI) scan. If the infant did not meet eligibility criteria at birth, the dyad was not enrolled in the study. A total of 203 mother-infant dyads were officially enrolled in the study, of whom n = 81 identified as BIPOC for inclusion in the current study, and n = 79 completed the Experiences of Racism Scale (REQ).

Due to in-person data collection restrictions due to the COVID19 pandemic, the current sample represents a sub-sample of the larger BEE Study. Two participants did not complete the second data collection session (neonatal MRI visit), and 22 participants completed the session remotely, thus 55 participants had an MRI scan. Of those who received an MRI scan, 22 participants were missing resting state fMRI data due to technical issues, including waking prior to the scan (the resting state fMRI was the last sequence collected in the imaging series); and 8 participants were excluded from analysis due to excessive motion during the MRI scan (described below), resulting in n = 25 participants in analyses reported here.

Pregnant participants (mean age = 28.5 years, SD = 6.2 years) identified as Black (n = 21), Asian (n = 1) and “Other” race (n = 3), including 2 individuals who identified as Hispanic (Table 1). Pregnant participants’ mean income-to-needs ratio was 2.54 (SD = 2.74), general stress from the Perceived Stress Scale (PSS) was 1.48 (SD = 0.65)^44^, and mean global severity index on the Brief Symptom Inventory (BSI) was 0.48 (SD = 0.49; Table 1)^45^. Data on maternal trauma history and/ or PTSD were not collected. Infants (n = 13 female; n = 12 male) were 13.5 days old, on average, at the time of the MRI scan (SD = 2.45, range = 10–20 days; Table 1).

**Table 1:** Participant demographic information.

Characteristic		Mean	SD	Minimum	Maximum
Infant gestational age at birth (days)		289.16	5.41	278	300
Infant chronological age at MRI (days)		13.54	2.45	10	20
Maternal income-to-needs ratio		2.54	2.74	0	11.28
Maternal BSI global symptoms index		0.48	0.49	0	1.70
Maternal perceived stress (PSS)		1.48	0.65	0.20	2.80
		Number		Percent	
Infant sex	Female	13		52	
	Male	12		48	
Maternal race	Asian	1		4	
	Black / African American	21		84	
	Other	3		12	
Maternal ethnicity	Hispanic	2		8	
	Not Hispanic	23		92	

*MRI* Magnetic resonance imaging, *BSI* Brief Symptom Inventory, *PSS* Perceived Stress Scale.

### Data collection

Pregnant participants completed a lab-based prenatal data collection visit during which they answered questionnaires, including demographics, income-to-needs ratio (INR), and the BSI^45^, among other measures collected to address aims of the larger project in which the current study was embedded^46^. When infants were approximately 2 weeks old participants completed a second study visit consisting of a neonatal MRI scan. At the end of each visit, mother-infant dyads were compensated with up to $125 in the form of a gift card, and a small gift of diapers and baby shampoo. All data included herein was collected prior to the pandemic-related shutdown in March 2020.

#### Maternal experiences of racism

Maternal experiences of racism were assessed at the prenatal visit using the REQ (Murry et al., 2001), which was adapted for its original use in African American populations to use across a diverse racial sample population, by changing “African American” to “race”. For example, instead of “because you are African American”, the adapted scale would read “because of your race”. The REQ is a standardized assessment that determines how often an individual has experienced different forms of racial discrimination over the past 5 years. It consists of 13 items, such as “How often have the police hassled you just because of your race?” that are rated on a 4-point scale (0 = *Never*, 1 = *Once or Twice*, 2 = *A few times*, and 3 = *Several times*). The first 11 items refer to direct, personal experiences with racism, and the last 2 items refer to racial discrimination experienced by the person’s friends and family based on their race. REQ items were summed and divided by the number of items (13) to calculate an average score. Higher scores indicate a higher number of racist experiences (α = 0.87 in the current sample; minimum = 0; maximum = 3). REQ scores were transformed with a natural log to correct for a highly skewed distribution, though results were consistent when using non-transformed REQ scores.

#### MRI acquisition

Images were acquired on a Siemens Prisma 3T scanner with a 32-channel head coil. Anatomical images consisted of a high-resolution 3D T2-weighted spin echo image (256 mm field of view [FOV], 0.8 × 0.8 mm isotropic resolution, TR/TE = 3200 ms/564 ms, 208 sagittal slices). Two, 6-min resting state functional MRI scans were acquired using a multiband, gradient echo EPI sequence (420 volumes, TR/TE/Flip = 800 ms/37 ms/52°, 208 mm FOV, 104 × 104 matrix, 2 × 2 mm in-plane resolution, 72 interleaved axial slices, 2 mm slice thickness).

### MRI processing

Functional images were processed with Analysis of Functional NeuroImages (AFNI)^47^ version 17.3, and the FMRIB Software Library (FSL), version 6.0.4 B-0 and fieldmap correction was performed using FSL’s topup^48,49^. Subsequent analysis steps performed in AFNI included: removal of the first 10 volumes; motion correction with 3dvolreg; bias field correction with 3dUnifize^50^; registration of the subject’s functional data to their anatomical image with 3dAllineate^51^. A 12-degree of freedom affine transformation was followed by nonlinear transformation using 3dNwarpApply to register each subject’s functional data to University of North Carolina (UNC) 0–1-2 Infant Atlases^52^, neonate space. Images were segmented into white matter, grey matter, and cerebrospinal fluid (CSF) with FSL’s FMRIB Automated Segmentation Tool (FAST) for use as masks that were eroded using a 3 × 3x3 voxel kernel and then used to generate ROI-averaged time series, with white matter and CSF time-series serving as nuisance regressors (along with their derivatives, the 6 motion regressors, global signal and its derivative) with 3dTproject. Time points where the volume-to-volume motion (Euclidean norm of the temporal differences of the 6 realignment parameters) exceeded 0.2 mm were censored during nuisance regression and replaced by interpolating from neighboring low-motion time points. Images were band-pass filtered from 0.01 to 0.10 Hz and smoothed with a 6-mm full-width half-maximum Gaussian kernel.

We extracted the time-series from right and left amygdala and hippocampus seeds based on the UNC 0–1-2 Infant Atlases^52^, neonate parcellation and concatenated across the two scan runs’ timeseries. We regressed each time-series (separately for each seed ROI) back onto each participant’s data using AFNI’s 3dDeconvolve, which also censored high-motion time-points (greater than 0.2 mm framewise displacement)^53^. Participants (n = 8) were excluded from analysis if they had less than 5 min of data due to more than 40% of data points censored for motion. Voxel-wise, whole-brain connectivity was assessed based on the Fisher-Z transformed correlation between the seed and every other voxel in the brain.

### Statistical analysis

We examined the contrast for the main effect of experiences of racism on seed-based connectivity using REQ scores as a continuous, voxel-wise regressor. All voxelwise analyses were thresholded at *p* < 0.05 controlling for family-wise error using threshold-free cluster enhancement with FSL’s Randomise^54^, and controlled for infant sex and gestational age at the 2-week MRI visit. Code for data processing and analysis can be found at the following location: https://github.com/KralTRA/BEE-resting-fMRI.

## Results

### Self-reported experiences of racism (REQ)

**Figure 1:**
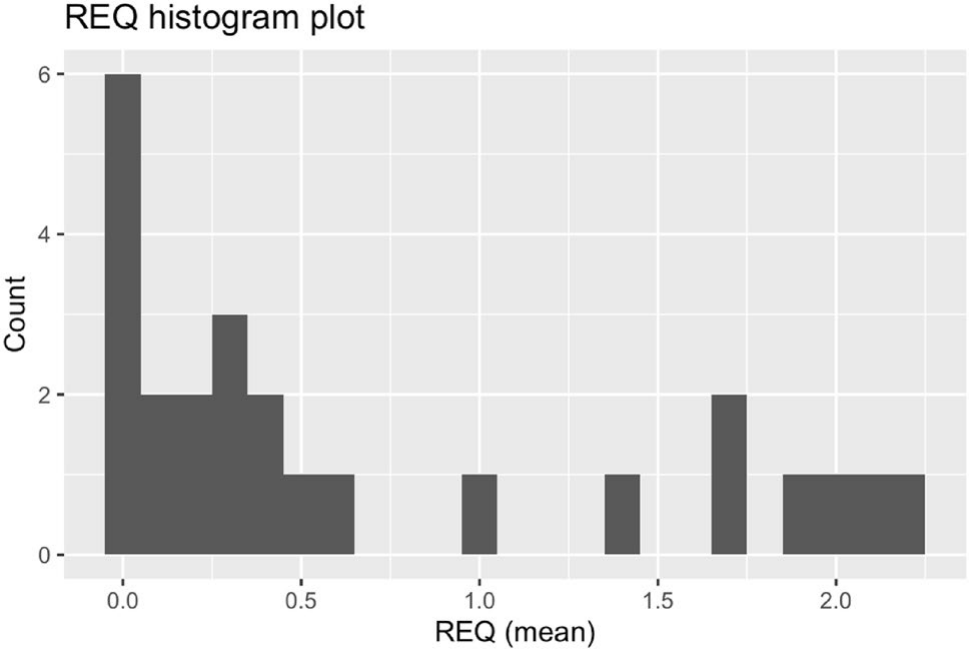
Histogram of self-reported experiences of racism (REQ).

Participants reported an average of 8.2 experiences of racism, with a range from 0 to 28 total experiences (Fig. 1). There was no relationship between REQ scores with infant sex (*p* = 0.89, b = 0.04, t(23) = 0.14) , or with gestational age (*p* = 0.30, b =  − 0.01, t(23) =  − 1.07). There was also no relationship between REQ scores and income-to-needs ratio (*p* = 0.29, b = 0.06, t(23) = 1.08, r =  − 0.13), nor with the PSS (*p* = 0.05, b = 0.38, t(23) = 2.09, r = 0.35), nor with BSI global severity index (*p* = 0.14, b = 0.18, t(22) = 1.54, r = 0.24).

### Resting state functional brain connectivity

Cluster details for significant results are presented in Table 2. Results are consistent when adding an additional covariate to control for motion, and the number of time-points censored for motion was unrelated to the independent variables (*p*s > 0.10). Statistical maps are available at the Open Science Framework at the following web address, labeled with the manuscript title: https://osf.io/wunrm/.

**Table 2:** Detailed information for significant clusters sized 50 voxels or larger.

Seed	Cluster region	Peak coordinates			Maximum	Size
		X	Y	Z	t-value	(voxels)
Right amygdala	Lingual gyrus	55	72	46	4.47	1267
	Right thalamus	53	61	47	5.09	471
Right hippocampus	Left rolandic operculum	77	59	56	4.75	7137
	Left parahippocampal gyrus	69	55	38	5.92	297
	Left precuneus	64	69	68	3.32	64
Left hippocampus*	Left calcarine cortex	69	74	48	4.83	1867
	Left paracentral lobule	63	62	66	4.04	722
	Right angular gyrus	52	71	59	3.56	138
	Left rolandic operculum	76	58	56	4.10	70
	Right superior temporal gyrus	41	67	55	3.62	63

Coordinates and details of significant clusters (*p* < 0.05).

*Cluster details for significant results at *p* < 0.02.

#### Amygdala connectivity

**Figure 2:**
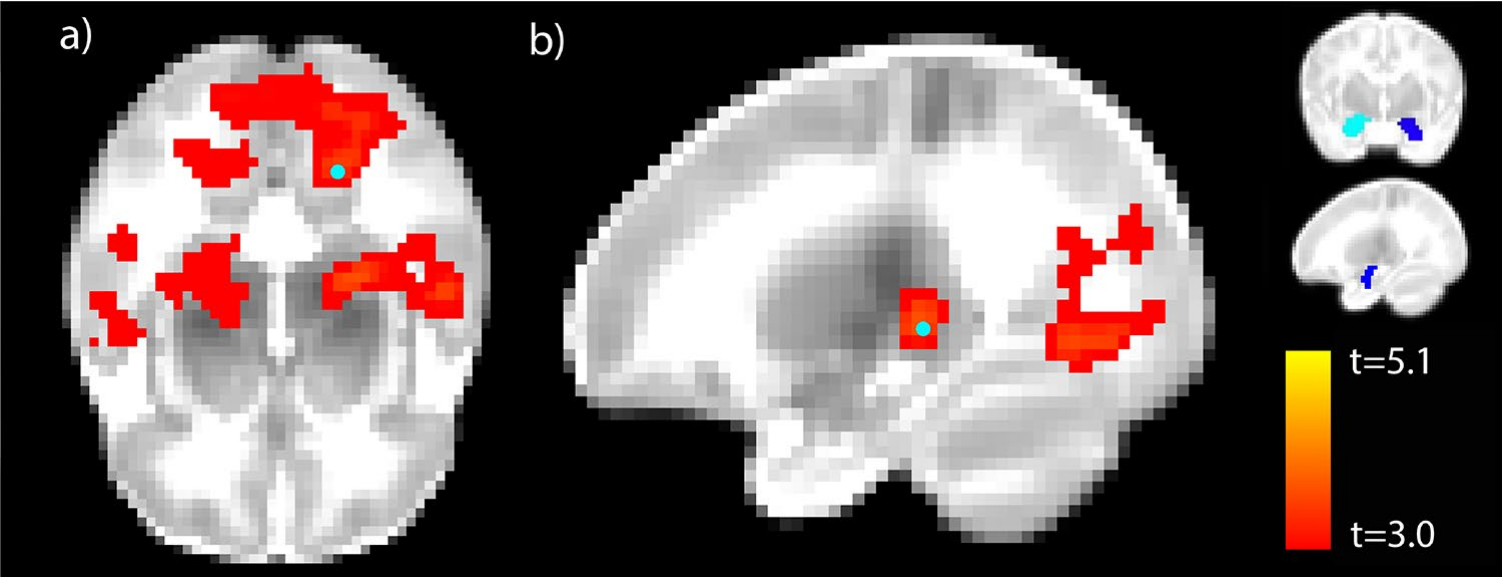
Higher right amygdala rsFC was associated with more experiences of racism. The peak of the visual cortex cluster (lingual gyrus) is overlaid in (**a**), and the peak of the thalamus cluster is overlaid in (**b**), both in teal circles (*p* < 0.05, maximum t-value = 5.1). Left and right amygdala seeds are depicted in light and dark blue, respectively (inset).

More maternal experiences of racism were associated with stronger right amygdala rsFC, with clusters in primary visual cortex and thalamus (Fig. 2) in a whole brain, voxel-wise analysis (*p* < 0.05, corrected). There was no significant relationship between REQ scores and left amygdala resting state connectivity, nor between INR, PSS, or BSI scores and either amygdala seed (*p* > 0.05), though the effects of REQ on amygdala rsFC were not significant when controlling for either INR or BSI scores. The effect of REQ on right amygdala rsFC remained significant when controlling for general stress with the PSS.

#### Hippocampus connectivity

**Figure 3:**
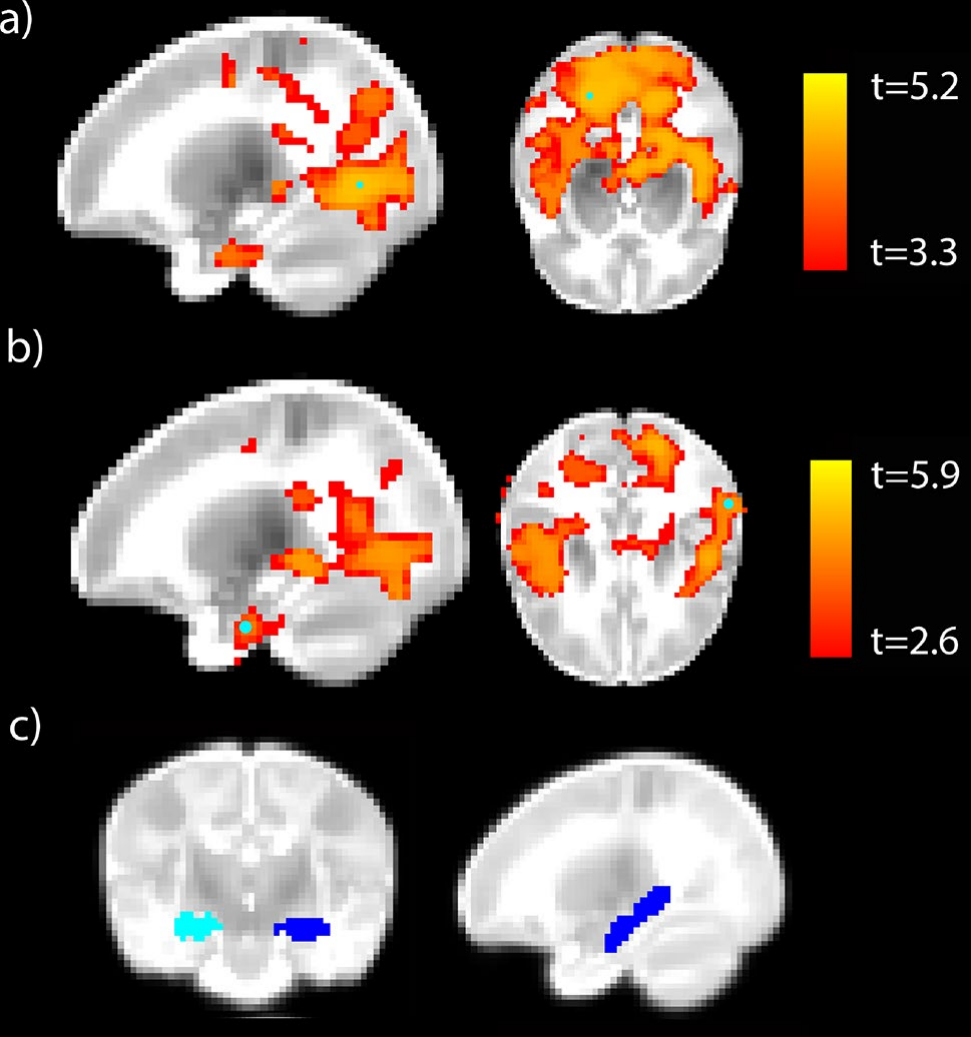
Increased hippocampus resting state functional connectivity (rsFC) was associated with more experiences of racism. The association of experiences of racism with (**a**) left hippocampus rsFC, and (**b**) right hippocampus rsFC. The peaks of the largest clusters are overlaid in teal circles (*p* < 0.05, maximum t-value = 5.9). Left and right hippocampus seeds are depicted in light and dark blue, respectively (**c**).

More maternal experiences of racism were positively associated with stronger left hippocampus rsFC with clusters in primary visual cortex (calcarine), middle and superior parietal cortex, and superior temporal cortex (Fig. 3a); and with stronger right hippocampus rsFC with clusters in superior temporal cortex, precuneus, and left parahippocampus (Fig. 3b), in a wholebrain, voxel-wise analysis (*p* < 0.05, corrected). The hippocampus regions of interest are depicted in blue (Fig. 3c). There was no relationship between INR, PSS, or BSI scores and hippocampus rsFC in this sample, though the effects of REQ on hippocampus rsFC were not significant when controlling for either INR or BSI scores. The effects of REQ on hippocampus rsFC remained significant when controlling for general stress on the PSS.

## Discussion

This study found preliminary evidence for intergenerational effects of the experience of racism on neonates’ functional brain connectivity—in networks associated with vigilance and emotional memory^55–58^. The more experiences of racism that BIPOC mothers reported, the stronger the neonatal functional connectivity of the amygdala and hippocampus to the primary visual cortex, among other regions. The current findings provide further evidence linking maternal health and well-being during pregnancy to neonatal outcomes. The results may suggest that maternal experiences of racism influence the infant brain to allow for adaptive coping to a threatening context, though such adaptations may become detrimental as individuals transition between different contexts, with differing needs for vigilance to threat. More likely, the impact of maternal experiences of racism on infant brain development is a maladaptive consequence, similar to the direct experience of trauma during childhood, which has known long-term, adverse outcomes for health and development^59–61^.

While research is scant regarding the developmental implications of alterations in neonatal amygdala—visual cortex rsFC, prior research has found that stronger neonatal amygdala rsFC with insula was associated with higher fearfulness at 6 months old^62^, and with depressive symptoms at 2 years old^26^, consistent with the known role of these neural circuits with anxiety and fear in adults^24,63^, and in older children with fear-based anxiety disorders^64^. Thus, we would hypothesize that increased neonatal amygdala—visual cortex rsFC may predispose individuals to higher vigilance in childhood, given the association of amygdala—visual cortex connectivity with vigilance, described above. Future research is needed to examine this hypothesis and further characterize the developmental implications of altered neonatal amygdala connectivity. Similarly, while few studies have examined neonatal rsFC in relation to maternal experiences of stress, our findings of stronger hippocampal-temporal rsFC are consistent with a recent study showing that higher maternal distress was correlated with stronger connectivity between the hippocampus and the temporal lobe in neonates, across multiple measures of distress (i.e., cortisol, perceived stress, pregnancy-related distress and depression)^43^. Suggesting that alterations in connectivity between these brain regions is shared across various measures and forms of distress, including experiences of racism. Other studies of prenatal stress suggest that the impact on the developing brain endures into childhood and likely beyond^65^, potentially increasing the risk for behavioral or psychiatric conditions later in development^66^. These specific links have yet to be tested directly in relation to maternal experiences of racial discrimination.

Understanding the parallels between racism and trauma, and forms of psychopathology like PTSD, has the potential to inform both preventative and secondary interventions for BIPOC individuals and communities aimed at negating the harmful effects of racism. Such efforts are critical to reduce individual suffering, yet they are insufficient to address the root cause of racism. If experiences of racism lead to heightened glucocorticoids in utero, e.g., similar to other forms of stress, then reducing racism-related stress and trauma for BIPOC mothers, is critical to ensuring health and flourishing in neonates. To intervene effectively, it is also vital to recognize the unique context of trauma induced by experiences of racism^67–70^, including its ongoing nature and pervasiveness, in the lives of BIPOC individuals.

This study was limited by a small sample size, which was primarily comprised of individuals who identified as Black, and future research should seek to replicate and extend these findings with a well-powered study, and to examine subsequent behavioral outcomes in infants and children. It is promising that the results seen here mirror what was previously shown in predominantly Hispanic mothers and their neonates’, whereby discrimination was associated with stronger amygdala rsFC to fusiform—another brain region involved in visual processing^14^. This study was also limited by the self-report modality of the REQ, which may be impacted by reporter and recall biases, as with all self-report measures. Six of the 25 participants reported zero experiences of racism (within the past 5 years), and it is unknown whether zero reported experiences of racism indicated a lack of occurrence, under-reporting (possibly due to the traumatic and stigmatic nature of the experience), or other phenomena.

Importantly, the results need to be replicated in future research powered to control for, and examine potential moderation by, factors such as poverty, stress and other forms of trauma. While we failed to find significant effects of INR and BSI on amygdala or hippocampus rsFC, these variables likely have overlapping variance with the REQ in its relationship to neonatal rsFC that the present study was underpowered to parse. Conversely, the effect of REQ was maintained when controlling for general stress assessed from the PSS, and the prior research in Hispanic women found evidence that discrimination is a distinct factor from other forms of stress^14^. A larger body of research indicates that stress and experiences of racism are independently associated with health outcomes, despite their known associations with each other and complex interactions in relation to some health-related variables^3^. Finally, the mechanism(s) by which racism-related stress and trauma in mothers may lead to neurodevelopmental differences in neonates warrants exploration and additional, hypothesis-driven research. Heightened glucocorticoids in utero provide one promising avenue for research^71^, and future studies should examine the interaction of changes in glucocorticoids and dynamics of trauma-related brain networks, such as the salience network. Animal studies examining the intergenerational transmission of stress-related experiences offer additional potential mechanisms. These studies show that both maternal and paternal experiences of stress alter DNA methylation and gene expression in genes that regulate maturation and arborization of neurons, as well as synaptic plasticity, which then impacts the cytoarchitectonics and connectivity of the developing offspring brain^72,73^.

In addition to the contributions of this study to developmental science, the results provide evidence for the critical need to address racism and provide support and resources for BIPOC communities^74^, and this work provides initial evidence that these impacts may extend beyond the mother, with downstream intergenerational effects that are already present in the neonatal brain, just after birth. In addition to addressing the negative impacts of racism on BIPOC individuals, it is critical to address racism directly, as a problem of White supremacist culture that is present across all socioeconomic strata and institutions of our society^75–79^. Future research should aim to replicate the current findings, and extend this work to examine the underlying biological mechanisms for intergenerational effects of prenatal stress, racism, and trauma, and to build off the impactful work of BIPOC scholars to test the efficacy of interventions to heal trauma from racism, e.g.,^80–85^.

## Data Availability

The datasets generated and analyzed during the current study are available from the corresponding author on reasonable request.

## Code availability

The custom code that was used for data processing and analysis in the current study is available via the project page on the Open Science Framework (https://osf.io/wunrm/).
