# Author Correction: Intergenerational effects of racism on amygdala and hippocampus resting state functional connectivity

**DOI:** 10.1038/s41598-024-70345-2

**Published:** 2024-08-20

**Authors:** T. R. A. Kral, C. Y. Williams, A. C. Wylie, K. McLaughlin, R. L. Stephens, W. R. Mills‑Koonce, R. M. Birn, C. B. Propper, S. J. Short

**Affiliations:** 1 Center for Healthy Minds, University of Wisconsin-Madison, Madison, WI USA; 2 Department of Psychiatry, University of Wisconsin-Madison, Madison, USA; 3 Department of Counseling Psychology, University of Wisconsin-Madison, Madison, USA; 4 Frank Porter Graham Child Development Institute, University of North Carolina at Chapel Hill, Chapel Hill, USA; 5 Department of Psychology and Neuroscience, University of North Carolina at Chapel Hill, Chapel Hill, USA; 6 Department of Psychiatry, University of North Carolina at Chapel Hill, Chapel Hill, USA; 7 School of Education, University of North Carolina at Chapel Hill, Chapel Hill, USA; 8 School of Nursing, University of North Carolina at Chapel Hill, Chapel Hill, USA; 9 Department of Educational Psychology, University of Wisconsin-Madison, Madison, USA

Correction to: *Scientific Reports* 10.1038/s41598-024-66830-3, published online 24 July 2024

The original version of this Article contained an error in the spelling of the author R. M. Birn which was incorrectly given as R. B. Birn.

The original Article has been corrected.

